# Evolution of the *sex*-Related Locus and Genomic Features Shared in Microsporidia and Fungi

**DOI:** 10.1371/journal.pone.0010539

**Published:** 2010-05-07

**Authors:** Soo Chan Lee, Nicolas Corradi, Sylvia Doan, Fred S. Dietrich, Patrick J. Keeling, Joseph Heitman

**Affiliations:** 1 Department of Molecular Genetics and Microbiology, Duke University Medical Center, Durham, North Carolina, United States of America; 2 Canadian Institute for Advanced Research, Department of Botany, University of British Columbia, Vancouver, Canada; 3 Institute for Genome Sciences and Policy, Duke University, Durham, North Carolina, United States of America; Tulane University School of Public Health and Tropical Medicine, United States of America

## Abstract

**Background:**

Microsporidia are obligate intracellular, eukaryotic pathogens that infect a wide range of animals from nematodes to humans, and in some cases, protists. The preponderance of evidence as to the origin of the microsporidia reveals a close relationship with the fungi, either within the kingdom or as a sister group to it. Recent phylogenetic studies and gene order analysis suggest that microsporidia share a particularly close evolutionary relationship with the zygomycetes.

**Methodology/Principal Findings:**

Here we expanded this analysis and also examined a putative *sex*-locus for variability between microsporidian populations. Whole genome inspection reveals a unique syntenic gene pair (*RPS9-RPL21*) present in the vast majority of fungi and the microsporidians but not in other eukaryotic lineages. Two other unique gene fusions (glutamyl-prolyl tRNA synthetase and ubiquitin-ribosomal subunit S30) that are present in metazoans, choanoflagellates, and filasterean opisthokonts are unfused in the fungi and microsporidians. One locus previously found to be conserved in many microsporidian genomes is similar to the *sex* locus of zygomycetes in gene order and architecture. Both *sex*-related and *sex* loci harbor TPT, HMG, and RNA helicase genes forming a syntenic gene cluster. We sequenced and analyzed the *sex*-related locus in 11 different *Encephalitozoon cuniculi* isolates and the sibling species *E. intestinalis* (3 isolates) and *E. hellem* (1 isolate). There was no evidence for an idiomorphic *sex*-related locus in this *Encephalitozoon* species sample. According to sequence-based phylogenetic analyses, the TPT and RNA helicase genes flanking the HMG genes are paralogous rather than orthologous between zygomycetes and microsporidians.

**Conclusion/Significance:**

The unique genomic hallmarks between microsporidia and fungi are independent of sequence based phylogenetic comparisons and further contribute to define the borders of the fungal kingdom and support the classification of microsporidia as unusual derived fungi. And the *sex*/*sex*-related loci appear to have been subject to frequent gene conversion and translocations in microsporidia and zygomycetes.

## Introduction

Microsporidia are obligate intracellular pathogens that mainly infect animals including fish and insects, and also some protists and crustaceans [1], [2]. In microsporidia, ∼150 genera and approximately 1,300 species are known [3]. Thirteen microsporidian species infect humans causing chronic diarrhea in immunocompromised individuals, mainly AIDS patients; in some cases, infection can also occur in otherwise healthy hosts [4]. Microsporidia have a uniquely specialized infection device called the polar tube [5]. The polar spore tube is coiled within dormant spores and when stimulation occurs by encountering and recognizing the host, the polar tube is everted and penetrates the host cell membrane. The polar tube then serves as a conduit for delivery of the infectious material, the sporoplasm. Microsporidial cells lacking a cell wall replicate inside the host and produce meronts, which eventually form mature spores that are released from the host.

The phylogenetic placement of microsporidia has long been debated [6], [7]. Originally, microsporidia were placed within an artificial group, the schizomycetes (reviewed in [7]). They were then long considered to be related to other spore forming parasites, but due to the perceived lack of mitochondria, microsporidia were eventually considered to be an ancient eukaryotic lineage [8], [9]. Phylogenetic analysis based on protein coding sequences then aligned the microsporidia within the fungal kingdom [10], [11], [12], [13], [14], [15], and the lack of mitochondria was soon undermined by the discovery of a reduced “mitosome” in microsporidia [8], [16]. In addition, recent findings that microsporidian genomes harbor two syntenic ribosomal genes (*RPL21* and *RPS9*) that are also syntenic throughout the fungi (with a few exceptions, such as *Schizosaccharomyces pombe*), supports a fungal origin. The *RPL21-RPS9* synteny is only found in microsporidia and fungi [17], [18] and not in other opisthokonts or other eukaryotic groups, where these genes are unlinked.

Within the fungi, molecular phylogenetic analyses have suggested a relationship between microsporidia and either ascomycetes and basidiomycetes [19], [20] based on some genes, or with zygomycetes based on other genes [21], [22]. A phylogenetic relationship with the zygomycetes is also supported by the findings that microsporidian genome architecture is similar to that of zygomycetes, and strongly differs from any other extant fungal phyla [17]. One of the shared loci is of particular potential interest because it may represent a *sex* locus in microsporidia. This microsporidian *sex*-related locus is highly similar to the *sex* locus of the zygomycetes: both contain genes for a triose phosphate transporter (TPT), a high-mobility group (HMG) protein, and an RNA helicase. In zygomycetes, the *sex* locus orchestrates and regulates sexual reproduction [17], [23], [24], [25], [26], [27]. This synteny is unique to microsporidia and zygomycetes and absent in other fungi whose genomes are available, including chytridiomycetes, ascomycetes, and basidiomycetes. Therefore, the microsporidian *sex*-related locus could be involved in an extant sexual cycle, if it is indeed homologous. Furthermore, several recent studies identified the existence of the meiosis specific genes for ‘core meiotic recombination machinery’ including Spo11, Rad50/Mre11, Dmc1, Rad51, and Mlh1 in the microsporidian genomes [17], [28], which could be an indication of the possible existence of sex in microsporidia [29], [30]. However, *bona fide* sexual development of microsporidia has not been reported although there are some observations and inferences about the possibility based on morphological approaches [31], [32].

Here, we have examined the genomic architecture of microsporidia to identify further conserved characteristics to help determine the relationship between microsporidia and other opisthokonts. We have identified unique genomic characteristics shared only in microsporidia and fungi rather than other lineages in the opisthokonts and more divergent eukaryotic lineages that contribute to define the boundaries of the fungal kingdom by features independent of sequence based phylogenetic approaches. We have also analyzed the *sex*-related locus from multiple isolates of *Encephalitozoon* species to test for the presence of idiomorphic HMG genes as observed in the zygomycete *sex* locus, in which divergent *sexP* and *sexM* genes are encoded by the *sex* locus of (+) and (−) mating type strains, respectively. Further, we analyzed the TPT, HMG, and RNA helicase genes to determine the phylogenetic relationship with the homologous genes of zygomycetes. We discuss the evolutionary trajectory of the *sex* locus within the basal fungal lineages for the zygomycetes and microsporidia.

## Results and Discussion

### Genome structure data support the Microsporidia as fungi

**Figure 1:**
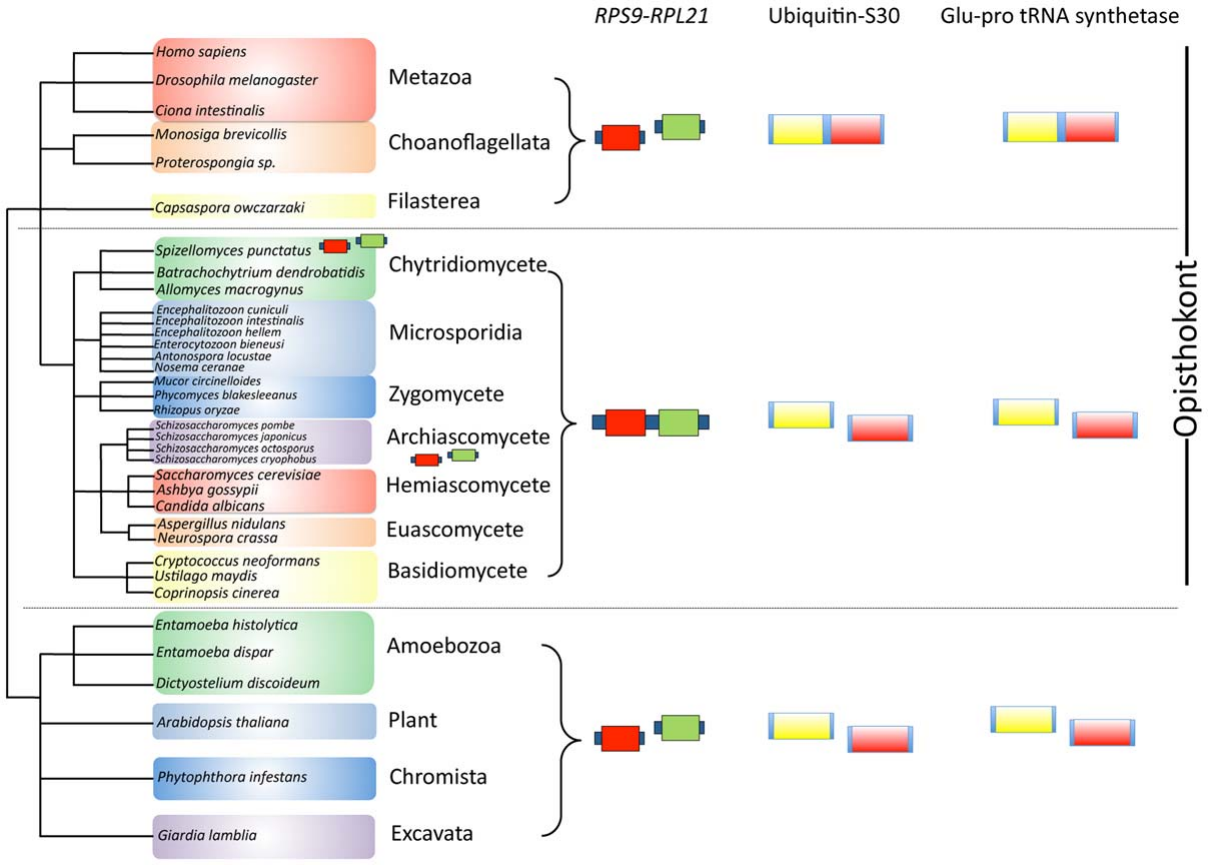
Unique genome characteristics for the fungi and microsporidia. The *RPS9-RPL21* synteny is uniquely found in the fungal and microsporidian genomes with two exceptions including *Schizosaccharomyces* species and *S. punctatus*. The two ribosomal genes, *RPS9* and *RPL21*, are present but unlinked in other non-fungal eukaryotes. Two gene fusions are present in the metazoan and pre-metazoan lineages. Ubiquitin and ribosomal small subunit S30 are encoded by one fused gene (ubiquitin-S30) and two amino-acyl synthetase domains for glutamine and proline are encoded by one gene (glu-pro tRNA synthetase). However, within the opisthokonts, the fungi and microsporidia do not contain either fused gene and two unlinked and separate genes encode each domain.

Virtually all available evidence now supports a phylogenetic relationship between microsporidia and the fungi, but whether they are within or sisters to the fungi remains contentious, as does exactly what fungal lineage they might be most closely related to. Individual gene phylogenies place the microsporidia close to the ascomycetes, basidiomycetes, or zygomycetes [19], [20], [22], [33], whereas combined tubulin phylogenies place them with the zygomycetes [21] and a four gene phylogeny suggests that microsporidia are related to *Rozella* [15]. We have found that microsporidian genomes share an overall higher degree of gene order conservation with zygomycetes than with any other fungal lineage that has been well sampled at the genomic level. Interestingly, one conserved gene pair found in microsporidia (*RPS9-RPL21*) was also identified in all known fungal genomes except that of *Schizosaccharomyces pombe* (Figure 1 and Figure S1) [17], [18], [34]. To test whether this gene pair is a stable marker for fungi, we searched more broadly within the fungi and other opisthokonts. Three other *Schizosaccharomyces* fission yeast species, *S. japonicus*, *S. octosporus*, and *S. cryophobus* were also found to lack this gene pair, and interestingly the chytrid *Spizellomyces punctatus* was also found to lack the *RPS9-RPL21* pair (Figure 1), and in these species homologs for both genes are present but unlinked. Thus, independent chromosome translocations might have occurred in the two fungal lineages that unlinked the two ribosomal genes in these exceptional species in the fungal kingdom.

Searching for similar genomic markers revealed two other highly conserved characteristics that help distinguish microsporidia from non-fungal opisthokonts. First, a gene encoding a fusion protein between ubiquitin and the ribosomal S30 protein is found in choanozoans as well as metazoans [35], whereas in all fungal lineages, including microsporidia, these two domains are present as separate genes that are unlinked (Figure 1 and Figure S2). Similarly, a glutamyl-prolyl tRNA synthetase fusion protein is also found in metazoans, choanoflagellates and the filasterean *Capsaspora* [36]. Here, two tRNA synthetase domains are linked by an RNA binding domain that regulates gene expression through controlling translation [37], [38]. We found no such fusion gene in the fungal genomes analyzed, including microsporidia, zygomycetes, chytridiomycetes, ascomycetes, and basidiomycetes (Figure 1 and Figure S3). Thus, these two fusion proteins appear to have arisen within the opisthokonts, after the divergence of the ancestors of fungi and metazoans. While these fusions do not suggest a specific fungal lineage that is particularly close to microsporidia, they do support the specific relationship between microsporidia and fungi as a whole to the exclusion of other major opisthokont lineages.

### Conservation of *sex*-related loci in microsporidia

**Figure 2:**
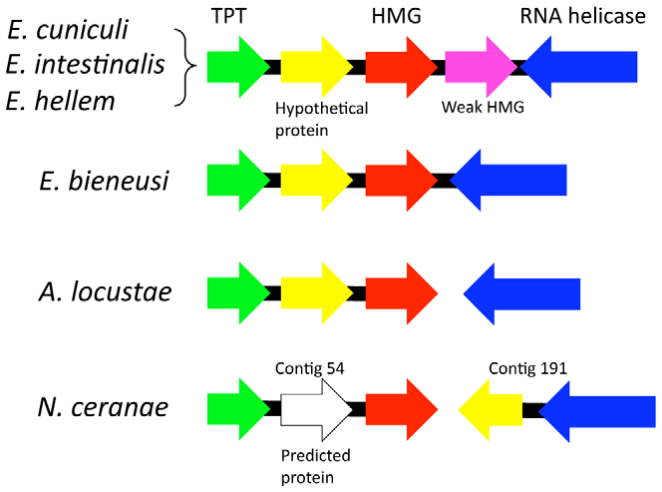
*sex*-related locus of microsporidia. *Encephalitozoon cuniculi*, *E. intestinalis*, and *E. hellem* share the same *sex*-related locus architecture containing the TPT, HMG, weak HMG, and RNA helicase genes. The weak HMG is not conserved outside *Encephalitozoon* species examined including *E. bieneusi*, *A. locustae*, and *N. ceranae*. In two insect pathogenic microsporidia, *A. locustae* and *N. ceranae*, the RNA helicase gene is unlinked to the HMG genes. The *N. ceranae sex*-related locus contains an additional predicted ORF between the TPT and HMG genes. A hypothetical protein is also linked in the *sex*-related locus across the microsporidia analyzed. Gene sizes are not to scale.

Among the many gene pairs found to be conserved between microsporidia and zygomycetes in our previous study, one of particular interest was a putative *sex*-related locus in microsporidia [39]. This locus, which comprises an HMG domain-containing gene flanked by RNA helicase and triose phosphate transporter (TPT) genes, is similar in architecture to the *sex* loci of the zygomycetes *Mucor circinelloides*, *Phycomyces blakesleeanus*, and *Rhizopus oryzae*. In microsporidia, an additional hypothetical protein is found downstream of the TPT gene, and the *E. cuniculi sex*-related locus also contains a second novel gene with limited identity to HMG genes (weak HMG protein gene) (Figure 2). Interestingly, the *sex*-related locus of the recently sequenced *Nosema ceranae* genome is similar to that of *Antonospora locustae* in that the RNA helicase gene is not linked to the HMG gene in either genome (Figure 2). Molecular phylogenetic analysis consistently shows *Encephalitozoon* is more closely related to *Nosema* than either are to *Enterocytozoon*, so this pattern suggests the locus was present in the ancestor of *Nosema*. Moreover, in *N. ceranae* a hypothetical protein that is found elsewhere in the genomes of all other microsporidia investigated is found in place of the hypothetical protein linked to the HMG gene. Overall, the greater diversity of this locus now apparent suggests that gene conversion and translocation may have occurred frequently around/within the *sex*-related locus in microsporidia. This variation is also found in the *sex* locus of three zygomycetes, where the orientation of the TPT and SexP/M genes differs and there is a repetitive element or additional ORF found in the *P. blakesleeanus* and *R. oryzae sex* locus, respectively [17], [23], [40].

### Variation in the putative *sex*-related locus of microsporidia

If this region is a *sex* locus in microsporidia, we might expect to find variation corresponding to distinct *sex* alleles. To test for the presence of such variation, we compared the sequences of *sex*-related locus genes for the presence of multiple paralogs, and also characterized the putative *sex*-related locus from three distinct species and multiple strains in the genus *Encephalitozoon*.

**Figure 3:**
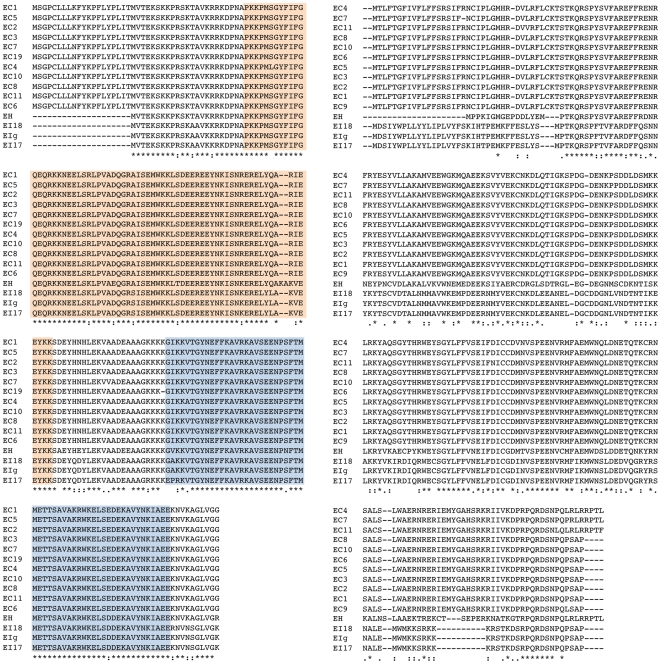
Alignments for HMG and weak HMG proteins in *Encephalitozoon* species. Alignment for the HMG proteins shows the HMG gene is highly conserved in eleven *E. cuniculi*, three *E. intestinalis*, and one *E. hellem* isolates suggesting divergent HMG genes are absent in this sample collection in contrast to the zygomycete *sex* locus which has *sexP* and *sexM* alleles. Tandem HMG domains are observed across the three species (highlighted boxes). Alignment for the weak HMG proteins also displays a relatively high level of conservation across the three species.

Characterization of the *sex*-related loci of *E. intestinalis* and *E. hellem* revealed an overall architecture identical to that of *E. cuniculi*: TPT, HP, HMG, weak HMG, and RNA helicase gene (Figure 2). The overall sequence conservation between homologous genes in these three species was also very high (for example see Figure 3). All *Encephalitozoon* isolates were found to be highly conserved (i.e., not an opposite mating type. See below) and the HMG proteins in all *Encephalitozoon* species as well as *A. locustae* have two HMG domains in the HMG protein (Figure 3) [17], whereas in the HMG proteins in *N. ceranae* and *E. bieneusi* only one HMG domain was found (data not shown) [17]. The HMG proteins have ∼90% identity between the three *Encephalitozoon* species, whereas the weak HMG domain proteins have relatively lower alignment scores (∼45% identity between *E. cuniculi* vs. *E. intestinalis*, ∼46% identity between *E. intestinalis* vs. *E. hellem*, and ∼51% identity between *E. cuniculi* vs. *E. hellem*). Comparison of the hypothetical proteins showed >78% identity between each species (data not shown). Thus, only the weak HMG domain protein was shown to be more diverged than the other proteins encoded by the *sex*-related locus between the three species. However the similarity is much higher than between SexP and SexM in zygomycetes. Given that the SexP and SexM proteins share only 14% and 20% similarity in *M. circinelloides* and *P. blakesleeanus*, respectively, the level of variation in microsporidian HMGs does not support the presence of idiomorphic HMG/weak HMG genes in the *Encephalitozoon* species complex.

To examine the level of variation within species for possible allelic heterogeneity, the *sex*-related locus was characterized from eleven genetically distinct *E. cuniculi* isolates and three strains of *E. intestinalis* from different host environments. The *sex*-related locus of the three *E. intestinalis* isolates was also very similar (>99% identity), as were all eleven isolates of *E. cuniculi*.

Given the level of sampling at least for *E. cuniculi*, it seems unlikely that multiple distinct *sex* type alleles exist with equal frequency at this locus, so if *E. cuniculi* sexual development occurs, is the *sex*-related locus involved and if so how? One possibility is that an opposite mating type allele/idiomorph is infrequent or rare, as is in case in the human pathogenic basidiomycete *Cryptococcus neoformans* [41]. A second possibility is that *E. cuniculi* is homothallic (self-fertile) [42] and sex might involve unisexual reproduction, similar to *C. neoformans* [43], [44], [45] or *Candida albicans* [46], [47]. The *E. cuniculi* HMG protein contains two HMG domains whereas zygomycete SexP and SexM have only one HMG domain (Figure 3) [17]. The two HMG domains may function separately and play the equivalent roles of SexP and SexM. This does not, however, explain the single HMG-domain containing proteins of *N. ceranae* and *Enterocytozoon bieneusi*. Alternatively, the weak HMG protein in the *Encephalitozoon sex*-related locus [17] (Figure 2) could play functions equivalent to SexP and SexM, but once again this protein was not observed in the other microsporidia.

### Linking the *sex*-related locus to sexual development in microsporidia

**Figure 4:**
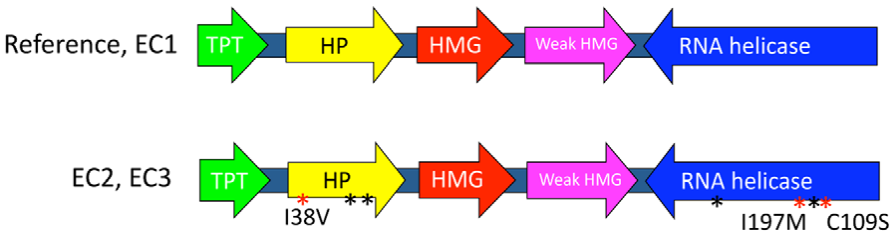
*sex*-related locus comparison between *E. cuniculi* isolates. The *sex*-related locus of EC1 and the reference isolate share 100% sequence identity, whereas the EC2 and EC3 *sex-*related locus has four synonomous (black asterisks) and three nonsynonomous (red asterisks) base substitutions (I38V in the hypothetical protein and C109S and I197M in the RNA helicase). These limited base changes do not support assignment as idiomorphs.

As of yet, there is no direct evidence linking the *sex*-related locus to sex determination or sexual reproduction in the microsporidia. The types of evidence that would be necessary to assign such a functional role could include a demonstration of opposite alleles or idiomorphs present in different isolates. We have sequenced the *sex*-related locus in eleven different *E. cuniculi* isolates, however, and found them to be highly conserved with the exception of a few point mutations, which tellingly are restricted to the flanking genes and are not found in the HMG or weak HMG genes (Figures 3 and 4).

Of course the zygomycete *sex* locus is just one strategy of many used in fungi. Given the independent data favoring a zygomycete origin of microsporidia, both from genome structure and some molecular phylogeny, it is the most reasonable system to search for initially, but others should also be considered if the *sex*-related locus proves not to be related to sex after all. For example, basidiomycetes utilize homeodomain (HD) transcription factors to orchestrate and regulate sexual development, such as the *Ustilago maydis b* alleles that encode two divergently transcribed HD1 and HD2 class proteins (reviewed in [48]), or the *Coprinopsis cinerea A* locus that also encodes two divergently transcribed homeodomain proteins [49]. In *Cryptococcus*, the α and **a** mating type locus encodes different classes of HD proteins, in which the *MAT* **a** allele encodes only an HD2 factor (Sxi2**a**) while the *MAT*α allele encodes only an HD1 factor (Sxi1α), in contrast to other basidiomycete *MAT* loci [50]. It would be worthwhile to investigate whether microsporidia have a pair of HD genes that are idiomorphic between isolates, as in *U. maydis*, or two functionally different HD in the same locus, as in different isolates as in *Cryptococcus*. We investigated three homeodomain gene clusters described previously (six HD genes: ECU03_0600, ECU03_0610, ECU04_0970, ECU04_1030, ECU10_1470, and ECU10_1480) [17], [51] in three *E. cuniculi* isolates that were previously proposed to represent candidate mating type loci [52]. Once again, however, no evidence for distinct alleles or idiomorphs was apparent from sequence analysis across the three homeodomain gene clusters (GenBank accession at HM049491 to HM049502) (Figure S4).

Although there is some morphological data that has been interpreted to suggest that some microsporidian species may undergo sexual reproduction [31], [32], this is not known for any of the six species in the current analysis. The type of evidence that would be necessary to show this definitively includes a demonstration of marker exchange (recombination) following co-infection of distinct isolates, or the finding that ploidy changes occur in the population (such as the finding of diploid or dikaryon isolates, or isolates heterozygous for genomic markers). Finally, to provide evidence linking the *sex*-related locus to sexual reproduction will require, for example, documentation that the genes therein are expressed at an appropriate time in the life cycle, or to show a candidate protein binds physically to the promoters for meiotic gene homologs by chromatin immunoprecipitation studies. These and other studies are ongoing to test whether sexual reproduction occurs in *E. cuniculi*, and whether the *sex*-related locus participates in this process.

### Oblique ortholog assignment between the *sex*-related/*sex* locus genes of microsporidia and zygomycetes

The *sex*-related locus in the *E. cuniculi* genome was originally identified using Blast searches with the *sexP* gene from the functionally-defined *sex* locus from *Phycomyces blakesleeanus* [23]. This revealed the homologous *E. cuniculi* HMG gene that is unique in the genome, and the region around this gene was then subjected to manual annotation and inspection, revealing the presence of a flanking triose phosphate transporter (TPT) gene and an RNA helicase gene homologs, strikingly similar to the organization of the *sex* locus in *P. blakesleeanus*, and also in *Mucor circinelloides* and *Rhizopus oryzae*. Here we address whether the genes flanking and within the *sex* and *sex*-related loci are orthologs or paralogs.

**Figure 5:**
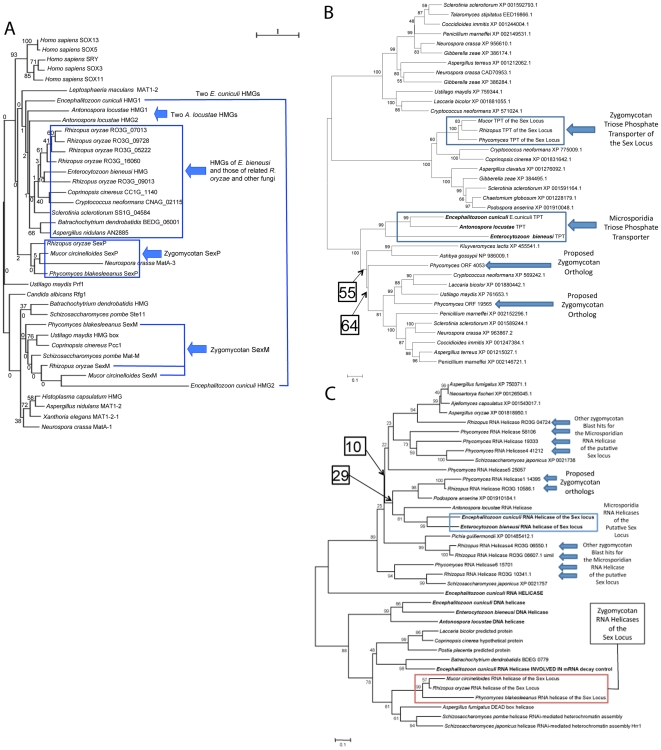
Phylogenetic analyses of the HMG, TPT, and RNA helicases. (A) One of the *E. cuniculi* HMG domains (HMG2) is aligned with SexM of the zygomycetes. The other HMG domain (HMG1) is aligned with HMG domains of zygomycetes other than SexP or SexM. The HMG domains of *E. bieneusi* and *A. locustae* are also aligned with HMG domains other than SexP or SexM. Note the low bootstrap values on each node of the tree. (B) The microsporidian TPTs are aligned to other TPTs rather than the one in the *sex* locus. This result suggests that the TPTs in the *sex*-related/*sex* loci are paralogs (for further discussion, see the text). (C) The microsporidian RNA helicases are also paralogs to the zygomycotan *sex* locus RNA helicases. Scale indicates an amino acid alteration per position (see the text for further discussion).

With respect to the HMG gene contained in the *sex*-related locus, while Blast searches with *P. blakesleeanus* SexP identify the *E. cuniculi* gene as homologous, reciprocal Blast searches with the *E. cuniculi* gene against other fungi return a variety of related HMG proteins in the *R. oryzae* genome. Construction of phylogenetic trees based on either the isolated HMG domains or the full-length proteins suggests that the high rate of divergence makes identifying orthologs problematic. Indeed, no microsporidian genes that are clear candidates to be orthologous with either SexP/M were found, although one domain of the *E. cuniculi sex*-related locus gene did branch with the *M. circinelloides* SexM with no bootstrap support. Further, there was low bootstrap support for virtually all nodes in the tree (Figure 4A). In fact, the zygomycete *sex* genes do not form a clade even though there is no question that the *sexM* and *sexP* genes are paralogous given the level of sequence identity and functional analysis (Figure 5A). Similarly, the microsporidian HMG domains (also including both domains from the dual-HMG genes) do not form a clade either, even though they are found in nearly identical genomic contexts and the orthology of the microsporidian TPT and RNA helicase genes is not in question (see below). In reciprocal Blast searches, there is a second HMG domain gene in the *P. blakesleeanus* genome that does share a modestly higher level of identity with the microsporidian HMG domain gene in the *sex*-related locus (jgi|Phybl1|79113|estExt_ fgeneshPB_pg.C_220136). The HMG domain gene family is very divergent and subject to accelerated rates of change in the case of the *sex* locus. In addition the evolutionary trajectory for an HMG domain determinant in the zygomycetes, which are heterothallic and have two opposite mating type genes (*sexM* and *sexP*), may be very different in a hypothesized homothallic species that may have a single sex determinant. It may simply be the case that this is an example of syntenic orthologs that do not share the highest level of identity with the syntenic partner, a problem for which there are precedents in other fungi.

We also performed phylogenetic analysis of the microsporidian TPT, which identified another gene in the zygomycete genomes that is more closely related compared to the *sex* locus linked TPT gene (Figure 5B). In maximum likelihood analysis, the zygomycotan *sex*-linked TPT genes were also closely related, so we find it hard to conclude with much certainty which of these genes are orthologous.

There are multiple paralogs of the RNA helicase present in microsporidian genomes, and again phylogenetic analysis reveals that the RNA helicase genes linked to the *sex* locus and the *sex*-related locus are also either paralogs or highly derived orthologs (Figure 5C). The microsporidian RNA helicases of the *sex*-related locus group in a clade that is distinct from the zygomycotan RNA helicases of the *sex* locus. However, within that clade, the support for the position of the proposed orthologs is extremely low. In fact, the entire clade is unsupported, so there is no evidence that the microsporidian helicases of the *sex-*related locus are more closely related to the ones proposed in Figure 5C than to any zygomycete helicase in this family. Overall, therefore, both the TPT and RNA helicase genes flanking the *sex* or *sex*-related locus are either highly derived orthologs or paralogs.

It is interesting to ask, if the RNA helicase and TPT genes are not orthologous to those in the zygomycete *sex* locus, why are the microsporidian, especially *E. cuniculi/intestinalis/hellem* and *E. bieneusi*, HMG genes also surrounded by a TPT and RNA helicase gene? The functional and genomic architecture of the microsporidian *sex*-related locus is highly similar to the zygomycotan *sex* locus, even though the sequences of the genes are highly derived. Moreover, this architecture (TPT/HMG/RNA helicase or TPT/HMG gene cluster) is conserved only between these two groups and is not shared with other fungal phyla or representative outgroups, including the choanoflagellate *Monosiga brevicollis* and the filasterea *Capsaspora owczarzaki* (see also reference [17]).

**Figure 6:**
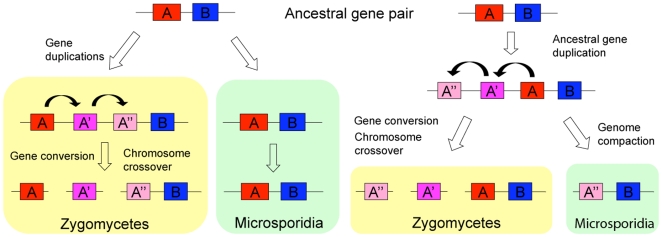
Hypothesis for the paralogous relationship between syntenic genes in zygomycetes and microsporidia. In the model presented, gene duplication, gene conversion, and chromosome translocation within the syntenic regions resulted in the formation of a syntenic locus with paralogous rather than orthologous genes.

The question, therefore, is whether there is a functional relationship between these three genes in the microsporidian *sex*-related locus and those in the zygomycete *sex* locus? There are several possible interpretations of these findings. First, the three genes may represent positional orthologs that are rapidly diverging as a result of gene duplication and conversion (Figure 6) and therefore appear less closely related. Gene duplications tend to occur locally, leading to linked paralogs. Gene loss can then result in paralog-ortholog syntenic gene pairs compared to ortholog-ortholog gene pairs. There are clear examples of this throughout the Ascomycota in which positional information has been necessary to correctly assign orthologous relationships [53]. Given the nature of the mating type locus and genes resident therein as rapidly evolving [54], [55], [56], [57], it is likely that similar criteria will be necessary here as well. There are also clear cases in hemiascomycetous fungi in which true orthologs can be defined by synteny, though they are not always the gene pair that shares the highest level of identity in pairwise comparisons of the two genomes. One striking example involves the six kinesin genes in *Saccharomyces cerevisiae* compared to *Ashbya gossypii*. There is another important example for this in the microsporidia. A cellular hallmark of all microsporidia is the presence of a polar tube. The proteins that compose the tube (PTP1, 2 and 3) are extremely divergent between members of the group, to the point that synteny is actually the only available way (outside antibody precipitation) to identify and annotate these proteins in newly sequenced genomes [58]. In *N. ceranae*, two of the PTP proteins only share 16.7% and 19.6% percent identity, respectively, when aligned with their putative *E. cuniculi* orthologs. Thus, it is simply not possible to assign the correct orthologous relationships by Blast searches, but syntenic position information can reveal which are orthologs based on conservation in the flanking genes [53]. Another striking example of difficulty in assigning orthologs based on sequence similarity is the case of the *BAR1* gene in *Candida albicans* and *S. cerevisiae*. The Bar1 protein encoded by *BAR1* is an aspartyl protease that degrades the alpha factor pheromone in *S. cerevisiae* [59]. In studies by Schaefer *et al*., Blast searches of the *C. albicans* genome with *S. cerevisiae* Bar1 revealed 14 homologous proteins, and the 12^th^ protein, based on the sequence comparison % similarity, was found to be the *bona fide* Bar1 ortholog based on functional studies. The 11 paralogs that share greater similarity than the *bona fide* ortholog are members of the SAP protease family [46], [60].

**Figure 7:**
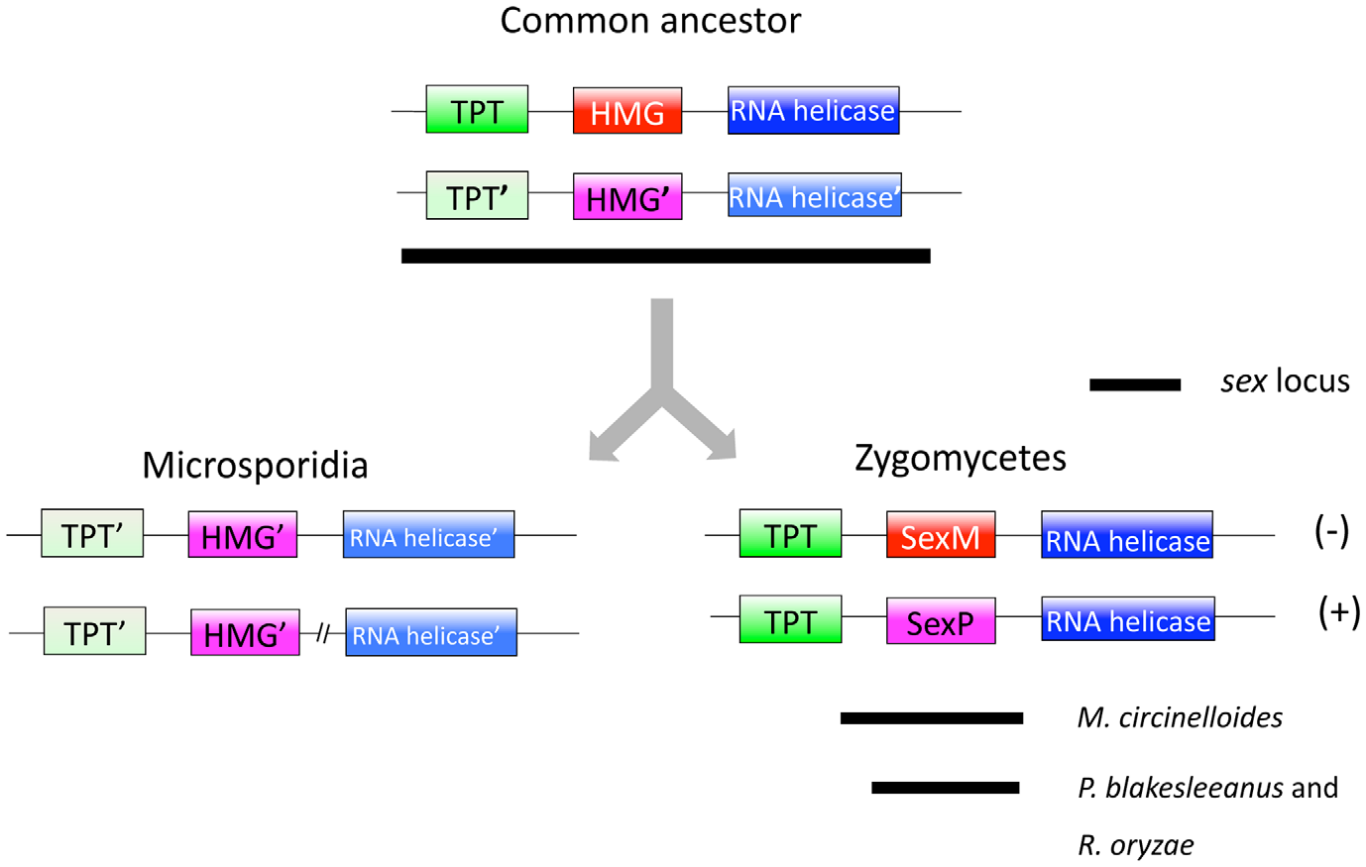
Evolutionary trajectory of the *sex* locus within zygomycetes and microsporidia. An ancestral *sex* locus might have spanned the TPT and RNA helicase genes. Thus, two divergent TPT, HMG, and RNA helicase genes were present in the two *sex*-alleles. In the microsporidian lineage, only one allele was retained. In the zygomycetes, local recombination might have fixed the TPT and RNA helicase genes resulting in one allele for these genes, whereas two alleles of the HMG genes remain in the two opposite mating types.

Second, the HMG genes may be orthologs and one or both of the flanking genes may share a paralogous relationship (see Figure 7). The promoter of the TPT gene is part of the *M. circinelloides sex* locus, indicating that these genes lie at the junction spanning both the common and diverged regions of the genome at the border of the *sex* locus. From detailed analyses of the mating type locus of *Cryptococcus*, it is clear that genes can be evicted from this locus, and genes which are quite divergent and clearly part of *MAT* are fixed as one of the two paralogs (*IKS1*, *NMC1*, *BSP3*) in other closely related lineages [61], [62]. Moreover, gene duplication and conversion events can occur within *MAT* which change the phylogenetic relationship of the resident genes (Figure 6). Thus, one possible scenario is that the TPT, HMG, and helicase genes were all part of an ancestral locus, and that one or the other paralog for the TPT and helicase gene was fixed and linked to the HMG gene in two descendent lineages. Sequence analysis of other basal fungi will allow this model to be tested in further detail. This notion has some interesting implications developed in more detail below (see Figure 7).

Third, the three genes of the *sex*-related locus identified in the microsporidia may each be paralogous with the genes in the *sex* locus of zygomycetes. This model is the most straightforward in terms of the available sequence data, but it invokes the convergent evolution of two similar gene clusters including a TPT transporter, an HMG domain protein, and an RNA helicase. Remembering that genome structure and some phylogenetic analysis both support a close relationship between microsporidia and zygomycetes [12], it seems unlikely that a gene cluster with such functional significance in zygomycetes would also assemble by chance independently in their sisters, the microsporidia (see supplementary discussion File S1).

### A model for the evolution of mating type loci by gene capture and eviction

A *P. blakesleeanus* TPT gene unlinked to the *sex* locus is present in the genome that may be more closely related to the TPT gene linked to the *sex*-related locus of microsporidia. Again this may simply be a case in which the gene is rapidly evolving and sequence similarity is not sufficient to correctly assign the orthologous relationship. However if we assume that the TPT genes linked to the *sex* and the *sex*-related locus are paralogous, it leads to an interesting model for the evolution of the mating type locus (Figure 7). The assignment of the *sex* locus in *P. blakesleeanus* and *M. circinelloides* has been definitively established based on functional approaches that show the *sex* genes control sexual identity and orchestrate sexual reproduction [23] (Lee, Gryganskyi, Li, Vilgalys, and Heitman, unpublished data). One interesting feature involves the border of the *sex* locus and the TPT gene [17]. While the TPT gene and its promoter flank the *sex* locus in *P. blakesleeanus*, the ORF of the TPT gene lies in the region flanking the *sex* locus in *M. circinelloides*, and the promoter lies within the *sex* locus, and is thus sexually dimorphic. Thus, the TPT gene straddles the border of the *sex* locus, similar to the amelogenin gene at the border between the pseudoautosomal and sex-specific regions of the mammalian sex chromosomes [63].

This raises the possibility that in a last common ancestor, and possibly also in some extant species, the TPT gene promoter including the ORF was a *bona fide* component of the *sex* locus (Figure 7). There are clear examples throughout the fungal kingdom of genes that have been captured into the mating type locus, lost from the mating type locus, or evicted from the mating type locus [64], [65], [66], [67], [68], [69]. A striking example of this last process is found in the detailed comparisons of the mating type locus in the *Cryptococcus neoformans* and *Cryptococcus gattii* lineages (serotypes A, D, and B that have diverged over ∼40 million years of evolution) [61], [62]. In serotypes A and B, three genes (*IKS1*, *NCM1*, and *BSP3*) are clearly part of the mating type locus, present in both the **a** and α alleles, and sex-specific. In contrast, in the serotype D *C. neoformans* var. *neoformans* lineage, these three genes have been evicted from *MAT* by a gene conversion event, and fixed as the α allele in the 3′ flanking region of the *MAT* locus. Thus, these three genes were within *MAT* in the last common ancestor, and remain within *MAT* in two extant lineages, but are no longer within *MAT* in a related diverged lineage.

Based on this analogy, it is possible that the TPT gene was part of an ancestral *sex* locus, but then evicted by similar gene conversion events (Figure 7). If one TPT allele was fixed in the zygomycete lineage as part of the *sex* locus and the other allele was fixed in the microsporidian lineage but evicted from *MAT*, then the observed situation would arise. To test this model additional genome or *sex* and *sex*-related loci must be characterized from zygomycete and microsporidian lineages, and these studies are in progress.

Based on this model, at one point there may have been that an ancestral *sex* locus that contained two HMG domain genes, similar to heterothallic to homothallic transitions that occur commonly in other fungi. One or the other was lost or transposed elsewhere in the genome in a transition to heterothallism. As of yet it is unknown how the two HMG domains in the microsporidia arose, but each of the two domains appears to be more closely related to SexP than to SexM of the zygomycetes. Thus, this might have resulted from a SexP-SexP gene fusion that subsequently underwent accelerated evolution, and its orthologous relationship to the ancestral gene is now less clear based solely on percent identity comparisons for genes in this large, divergent family of transcriptional regulators.

Currently these comparisons are restricted to speculation by the paucity of genomic information from basal fungal lineages, currently limited to three Mucorales and two chytrid species, and from microsporidia, currently limited to three genomes (*E. cuniculi*/*intestinalis*/*hellem*, the last two of which are in progress to be released) and genome survey/draft genomes (*A. locustae*, *N. ceranae*, and *Octosporea bayeri*) [39], [70], [71], [72]. As additional whole genome analyses are reported these hypotheses can be tested more directly, and it is also possible that species with currently unsampled genomes may emerge as even more closely related to the microsporidia than are the Mucorales.

## Materials and Methods

### *E. cuniculi* cultures and genomic DNA extraction

Three *E. cuniculi* strains, EC1, EC2, and EC3 (Table 1), were kindly provided by Dr. Louis Weiss. These strains were isolated from the kidney of an infected rabbit, mouse, and dog, respectively and represent three genotypes of *E. cuniculi* [73], [74]. The strains were maintained in RK13 (rabbit kidney) cells. RK13 cells were grown in MEM media containing 7% FBS supplemented with penicillin-streptomycin (Invitrogen, Co.) as described previously [75]. Monolayers of RK13 cells were subject to *E. cuniculi* infection. The MEM media was changed twice a week and spent media was collected to accumulate spores. Genomic DNA from EC1, EC2, and EC3 was extracted as described [75]. *Encephalitozoon intestinalis* and *Encephalitozoon hellem* (Table 1) previously harvested from humans were maintained in RK13 cells [74], [76], [77]. Spores were isolated by sequential washing with dH_2_O, TBS-Tween 20 (0.3%), and TBS, followed by Percoll™ centrifugation, a final wash with TBS-SDS (0.1%), and genomic DNA was extracted using established protocols [78].

**Table 1:** Strains used in this study.

Isolate designation	Species	genotypes	hosts	references
EC1	*E. cuniculi*	genotype I	rabbit	[74]
EC2	*E. cuniculi*	genotype II	mouse	[74]
EC3	*E. cuniculi*	genotype III	dog	[74]
EC4	*E. cuniculi*	genotype III	dog	[74]
EC5	*E. cuniculi*	genotype I	rabbit	[74]
EC6	*E. cuniculi*	genotype II	mouse	[74]
EC7	*E. cuniculi*	genotype III	dog	[74]
EC8	*E. cuniculi*	genotype I	dwarf rabbit	[74]
EC10	*E. cuniculi*	genotype II	mouse	[74]
EC11	*E. cuniculi*	genotype III	human	[74]
EC19	*E. cuniculi*	genotype I	rabbit	[74]
EI17	*E. intestinalis*		human feces	[76]
EI18	*E. intestinalis*		human nasal	[76]
EIg	*E. intestinalis*		human alveolar	[76]
EH	*E. hellem*		human eye	[77]

### Identification and sequencing of the *sex*-related locus

The *sex*-related locus from the EC1, EC2, and EC3 isolates was amplified with high fidelity *Taq* polymerase (Roche) using primers, JOHE20578- CCGGTGTTCATCCTTCTGTT and JOHE20579-GCACGTCTCACAGTTGACCA. The amplicons were sequenced with primers JOHE20578, JOHE20579, JOHE20720- CAGTAAAAAGGCGCAAGGAC, and JOHE20721-CTAGAATGCGCCCCATACAT. Three independent PCR reactions were performed and analyzed. The *sex*-related locus of *E. intestinalis* and *E. hellem* was identified through in-depth genome surveys. This locus was samples in all isolates analyzed here using the following “forward” primer sets: TPTF1-TGTGCAGAGTTTACGCTCGTT; TPTF2-TGAACTATGTTGGGCTGACCATAA; TPTF3-CTGACCATAAGCATTGCAGGAAT in combination with the following reverse primers: HelicaseR1-GGCTTGACATTCCGTATCTCGAC and HelicaseR2-TACGACCTATGTGACGAGAGAACAT

The *sex*-related locus of *N. ceranae* and *RPL21-RPS9* synteny were identified by Blast with the *E. cuniculi* TPT, HMG, and RNA helicase genes using Formatdb against a local copy of the NCBI nr database.

### Annotation and phylogenetic analyses

Sequences were annotated by using FGENESH or ORFfinder (NCBI). For phylogenic analyses for the *sex*-related locus components, deduced amino sequences were aligned with ClustalW and the alignment results were manually inspected and corrected if needed. Maximum likelihood trees (bootstrap number  = 100) were generated by using the PhyML 3.0 software [79].

### Blast search for the *RPS9-RPL21* gene synteny, glutamyl-prolyl tRNA synthetase gene, and ubiquitin-ribosomal subunit S30 gene

Blast search with glutamyl-prolyl tRNA sythetase and ubiquitin-ribosomal S30 genes against *Mucor circinelloides* (http://genome.jgi-psf.org/Mucci1/Mucci1.home.html), *Phycomyces blakesleeanus* (http://genome.jgi-psf.org/Phybl1/Phybl1.home.html), *Rhizopus oryzae* (http://www.broadinstitute.org/annotation/genome/rhizopus_oryzae/MultiHome.html), *Batrachochytrium dendrobatidis* (http://www.broadinstitute.org/annotation/genome/batrachochytrium_dendrobatidis/MultiHome.html), *Allomyces macrogynus* (the Origin of Multicellularity Project at the Broad Institute), *Encephalitozoon cuniculi* (NCBI), *Enterocytozoon bieneusi* (NCBI), *Nosema ceranae* (NCBI), and *Antonospora locustae* (http://gmod.mbl.edu/antonospora) genomes was conducted to identify homologs in the basal fungal lineages. The flanking areas were manually annotated to test whether fused amino-acyl synthetases were conserved in these fungi. A synteny search for *RPS9-RPL21* was conducted against the genomes of *Capsaspora owczarzaki*, *Proterospongia* sp, and *Spizellomyces punctatus* presented in the database for the Origin of Multicellularity Project at the Broad Institute. For other eukaryotic lineages, *Giardia lamblia* (Excavata) (NCBI), *Phytophthora infestans* (Chromista) (Broad Institute), *Arabidopsis thalina* (Planta) (NCBI), and three amoebozoans (NCBI) including the *Entamoeba histolytica*, *E. dispar*, and *Dictyostelium discoideum* genomes were analyzed. Four archiascomycete genomes for *Schizosaccharomyces pombe*, *S. japonicus*, *S. octosporus*, and *S. cryophobus* (http://www.broadinstitute.org/annotation/genome/schizosaccharomyces_group/MultiHome.html) were analyzed for *RPS9-RPL21* gene synteny.

## Supporting Information

File S1 Calculation of the probability of convergence to similar gene clusters.
(0.04 MB DOC)

Figure S1 Fungal specific *RPL21-RPS9* gene cluster in newly sequenced microsporidians and fungi.
(0.34 MB TIF)

Figure S2 A fusion gene for ubiquitin and ribosome small subunit S30 found within non-fungal lineages in opisthokonts.
(0.36 MB TIF)

Figure S3 A fusion gene for two tRNA synthetases found within non-fungal lineages in opisthokonts.
(0.38 MB TIF)

Figure S4 Sequence comparison for homeodomain gene clusters in four *E. cuniculi* isolates.
(0.07 MB TIF)
