# Supplementary material for: Evolution of the sex-Related Locus and Genomic Features Shared in Microsporidia and Fungi
Source: PLoS One. 2010 May 7;5(5):e10539. doi: 10.1371/journal.pone.0010539 (PMC2866331; doi:10.1371/journal.pone.0010539)
Supplement: File S1 — Calculation of the probability of convergence to similar gene clusters. (0.04 MB DOC) [file pone.0010539.s001.doc]

**Supplementary discussion**

**Calculating the probability of convergence to similar gene clusters indicates this is unlikely due to chance**

Is the striking similarity observed in the gene content and organization of the *sex* and *sex*-related locus ancestral? At the root, the question raised is whether the *sex*-related locus and other syntenic gene clusters are ancestral or convergent. Because of variable rates of evolution orthologs and paralogs may be very difficult to assign in the mating type locus, because of WGD and other duplications, and because of the genome reduction and rampant divergence in the microsporidia. It is very hard to conceive how these genomic structures could have arisen via convergence. For example, we can think of as to why TPT, HMG, and RNA helicase genes should have independently converged to be linked in both the Zygomycota and the microsporidia, and then for the order of these genes to be highly conserved within these two groups. There are very few examples of conserved gene clusters in fungi, and these invariably involve mating type loci, natural product biosynthetic clusters, and a few rare examples of metabolic gene clusters. Thus, the finding that three genes of the same functional types are similarly organized in both lineages is, in and of itself, of considerable potential significance.

To make convergence more parsimonious, the probability of the *sex*-related gene cluster arising by chance in the microsporidian genomes was calculated. To gauge the likelihood that such a genomic organization could have occurred solely by chance in the microsporidian species, we calculated the probability of finding a TPT transporter in the -2, -1, +1, or +2 positions relative to the HMG domain gene, and then linked to an RNA helicase gene in one of the remaining positions. There are 1,998 defined genes in the *E. cuniculi* genome, and there is one TPT transporter gene, one HMG domain gene, and a series of RNA helicase orthologs and paralogs (~17). If the HMG gene is fixed at a position in the genome (its probability of being at a site in the genome is 1), the probability of the TPT gene being located within two genes of the HMG gene on either side at this position is 4/2000 (1/2000 for each of the four possible positions). Next the probability of finding one of the possible RNA helicase genes within the three remaining positions in a five-gene cluster similar to the observed *sex*-related locus can be calculated as is 3/2000 X 17, or 51/2000. The overall probability of finding the three genes, TPT-HMG-RNA helicase, in this arrangement is then the product of 4/2000 and 51/2000, or ~1/20,000. Thus, the odds of finding this particular gene cluster by chance in two divergent microsporidian genomes are exceedingly small, providing evidence against models in which these two gene clusters arose by a chance convergence. We should point out that this calculation is based on the very small number of proteins in the *E. cuniculi* genome, and the number of proteins in the ancestor of *A. locustae*, *E. cuniculi*, and *E. bieneusi* may have been much higher, which would further reduce the likelihood of this arrangement appearing by chance. However, it remains theoretically possible that there is some strong selective pressure leading to these genes being clustered independently in two divergent lineages, but we submit that this is not a very satisfying explanation as it lacks any biological basis. In contrast, our conclusion that the lineages are related is relatively straightforward, and has been proposed before based on molecular phylogenies.
