# Supplementary figures and images for: Evolution of the sex-Related Locus and Genomic Features Shared in Microsporidia and Fungi
Source: PLoS One. 2010 May 7;5(5):e10539. doi: 10.1371/journal.pone.0010539 (PMC2866331; doi:10.1371/journal.pone.0010539)

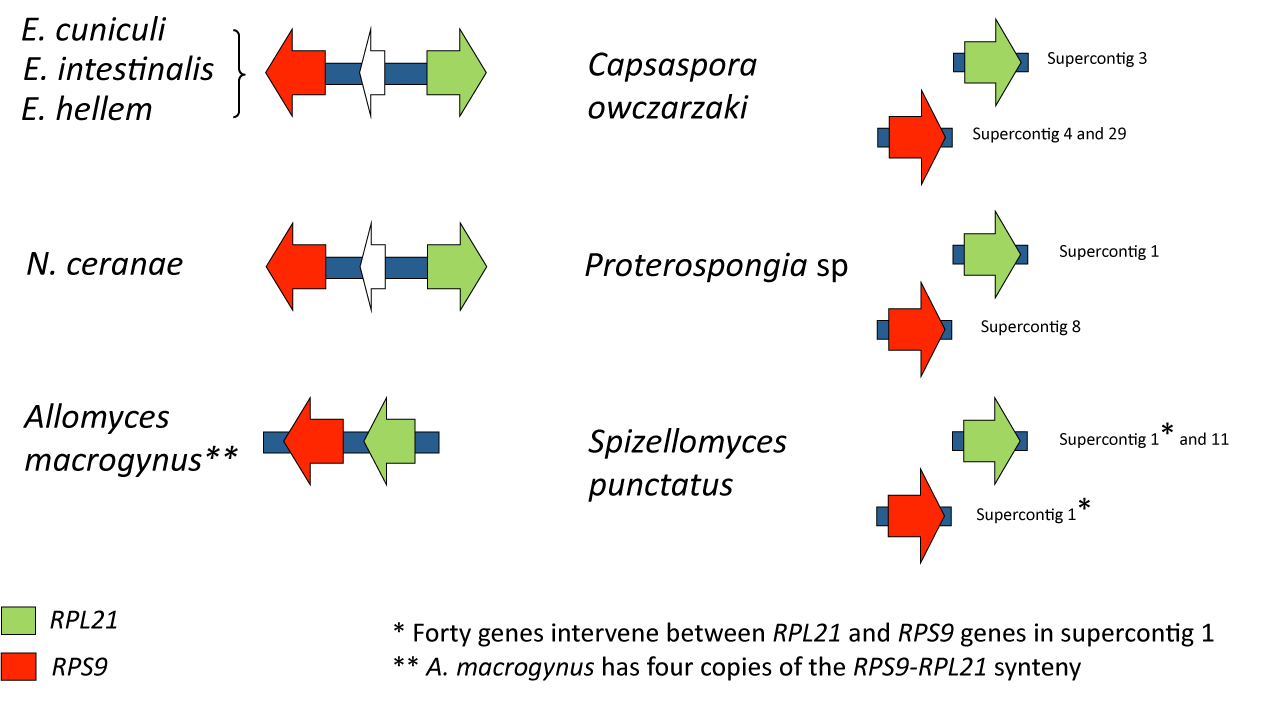

Supplement: Figure S1 — Fungal specific RPL21-RPS9 gene cluster in newly sequenced microsporidians and fungi. (0.34 MB TIF) [file pone.0010539.s002.tif]

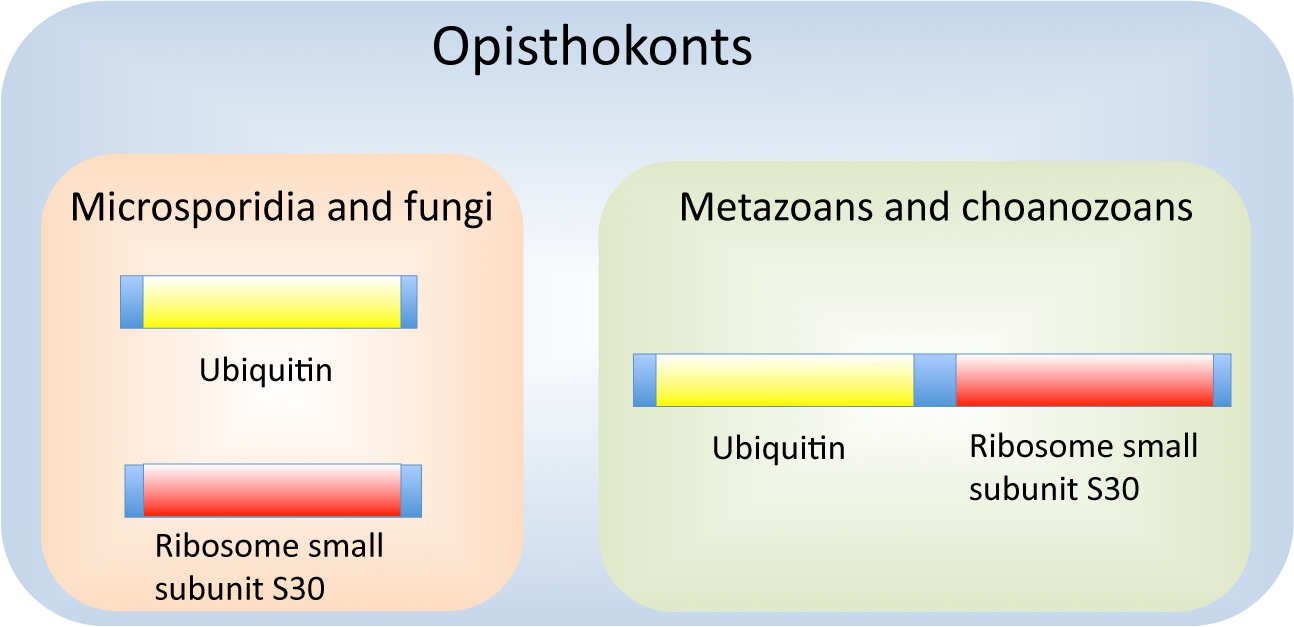

Supplement: Figure S2 — A fusion gene for ubiquitin and ribosome small subunit S30 found within non-fungal lineages in opisthokonts. (0.36 MB TIF) [file pone.0010539.s003.tif]

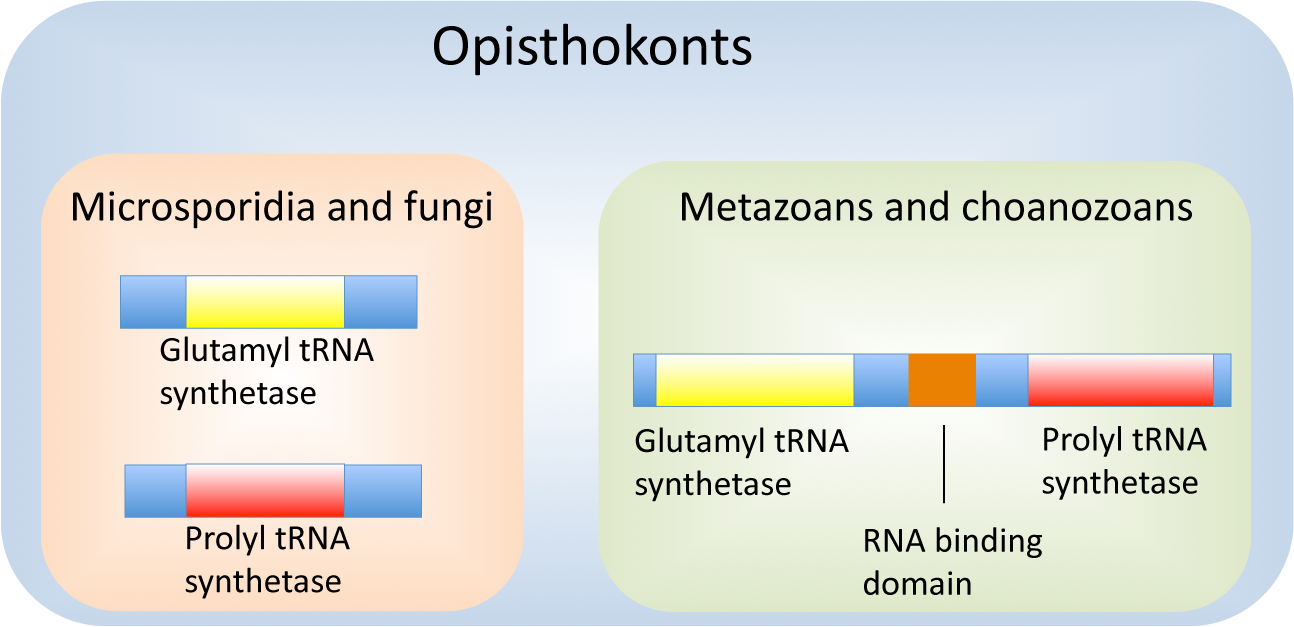

Supplement: Figure S3 — A fusion gene for two tRNA synthetases found within non-fungal lineages in opisthokonts. (0.38 MB TIF) [file pone.0010539.s004.tif]

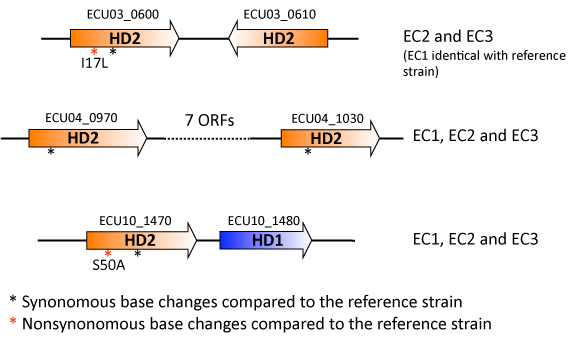

Supplement: Figure S4 — Sequence comparison for homeodomain gene clusters in four E. cuniculi isolates. (0.07 MB TIF) [file pone.0010539.s005.tif]
